# Supplementary material for: Endurance training increases a ubiquitylated form of histone H3 in the skeletal muscle, supporting Notch1 upregulation in an MDM2‐dependent manner
Source: J Physiol. 2025 Sep 5;603(19):5477–508. doi: 10.1113/JP288947 (PMC12487604; doi:10.1113/JP288947)
Supplement: Supplementary file 2 — Supp data [file TJP-603-5477-s001.pdf]

Figure 1.

Gastrocnemius muscle (Fig.1A-E):protein extraction was performed on gastrocnemius muscles. For PECAMblot, a protein extract from HDMEC(human dermal microvascular endothelial cells) wasused as a positive control. There was a weak and uneven TUBULINfor the original PECAM blot. Therefore, the PECAM blot was run twice and the values used for statistical analysis was the average values from the two blots.

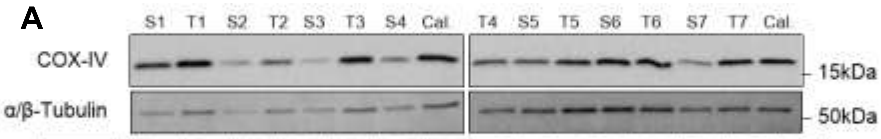

	S1	T1	S2	T2	S3	T3	S4	T4	S5	T5	S6	T6	S7	T7
COXIV/ α/β-Tubulin	0.69	1.60	0.26	0.63	0.90	1.50	0.25	1.03	0.44	0.65	1.28	1.13	0.48	1.85

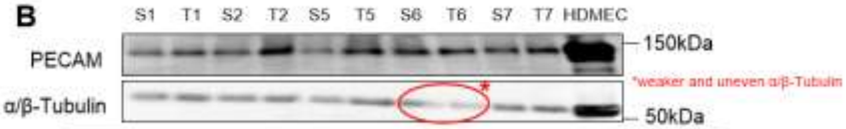

	S1	T1	S2	T2	S5	T5	S6	T6	S7	T7	HDMEC
PECAM/ α/β-Tubulin	0.10	0.13	0.11	0.24	0.10	0.17	0.27	0.37	0.14	0.18	1.09
Standardized to HDMEC	0.09	0.12	0.10	0.22	0.09	0.16	0.25	0.34	0.13	0.17	1.00

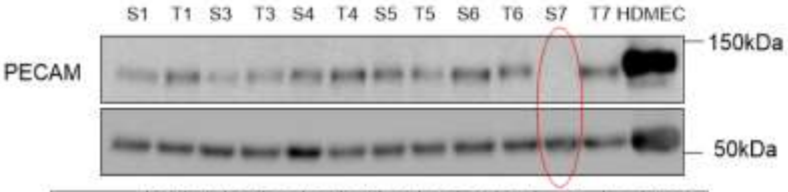

	S1	T1	S3	T3	S4	T4	S5	T5	S6	T6	S7	T7	HDMEC
PECAM/ α/β-Tubulin	0.31	0.50	0.20	0.35	0.25	0.73	0.39	0.35	0.49	0.48	No band detected	0.70	2.90
Standardized to HDMEC	0.11	0.17	0.07	0.12	0.09	0.25	0.13	0.12	0.17	0.17	-	0.24	1.00

	S1	T1	S2	T2	S3	T3	S4	T4	S5	T5	S6	T6	S7	T7	HDMEC
Blot 1: PECAM/ α/β-Tubulin	0.10	0.13	0.11	0.24	-	-	-	-	0.10	0.17	0.27	0.37	0.14	0.18	1.00
Blot 2: PECAM/ α/β-Tubulin	0.11	0.17	-	-	0.07	0.12	0.09	0.25	0.13	0.12	0.17	0.17	No band detected	0.24	1.00
Average	0.10	0.15	0.11	0.24	0.07	0.12	0.09	0.25	0.12	0.15	0.17	0.17	0.14	0.21	1.00

Plantaris muscle (Fig.1C-D): protein extraction was performed on all plantaris muscles. For PECAM an protein extract of HDMEC (human dermal microvascular endothelial cells) was used as a positive control.

C

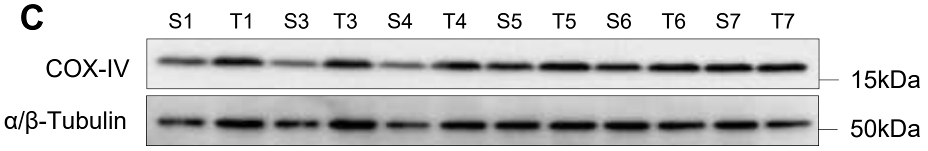

	S1	T1	S3	T3	S4	T4	S5	T5	S6	T6	S7	T7
COXIV/ α/β-Tubulin	1.16	1.22	0.93	1.14	1.04	1.36	1.21	1.46	1.18	1.50	1.40	1.61

D

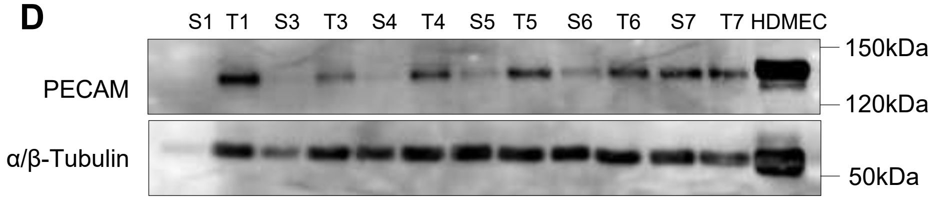

EDL muscle (Fig.1E): Representative immunohistochemistry staining of WGA and isolectin in sedentary and trained mice extensor digitalis longus (EDL) muscles. Scale bars represent 50 µm.

E

Capillar:Fiber ratio		CD (mm2)		Fiber density (mm2)	
Sedentary	Trained	Sedentary	Trained	Sedentary	Trained
1.00	1.28	301.91	463.19	302.35	363.23
0.93	1.14	402.79	351.50	431.08	343.00
1.00	1.29	302.17	527.95	306.38	409.50
1.14	1.14	437.86	351.50	394.12	306.62
0.88	1.08	425.77	540.81	481.53	499.85
1.03	1.36	444.28	335.40	429.57	247.30

Figure 2. Images of Immunoblots (IB)

Gastrocnemius muscle (Fig.2A-D): protein extraction was performed on gastrocnemius muscles. For EZH2, samples were loaded/distributed on two separate membranes. A calibrator (pooled protein sample) was used to standardize the ratios for data analysis.

A

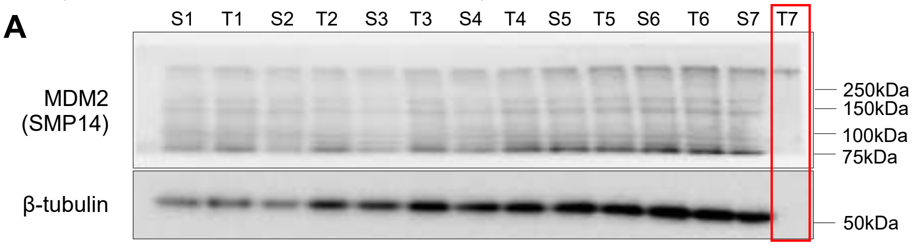

	S1	T1	S2	T2	S3	T3	S4	T4	S5	T5	S6	T6	S7	T7
MDM2 (SMP14)/ β-tubulin ratio	0.48	0.67	0.32	0.76	0.39	0.59	0.48	0.70	0.64	0.75	0.60	0.68	0.66	No band detected

Sample T7 did not run/transfer properly, therefore, this sample was excluded from data analysis.

B

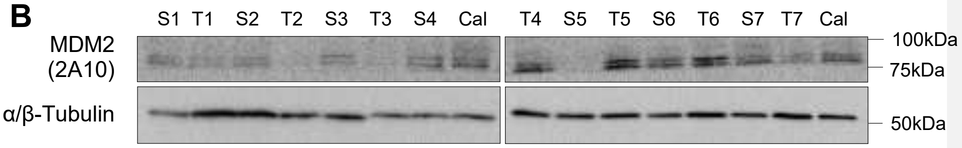

	S1	T1	S2	T2	S3	T3	S4	T4	S5	T5	S6	T6	S7	T7
MDM2 (2A10)/ α/β-tubulin ratio	0.95	1.13	0.34	0.56	0.35	0.82	0.73	1.27	0.16	0.96	0.73	1.24	0.82	0.82

C

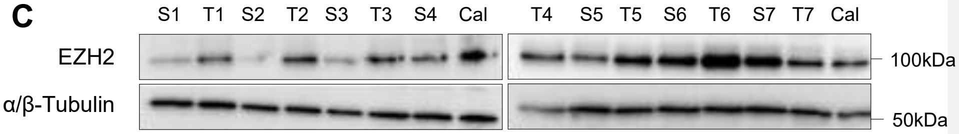

	S1	T1	S2	T2	S3	T3	S4	T4	S5	T5	S6	T6	S7	T7
EZH2/ α/β-tubulin ratio	0.44	1.04	0.32	1.73	0.90	1.60	1.07	1.18	0.53	1.00	1.23	1.78	1.17	0.72

D

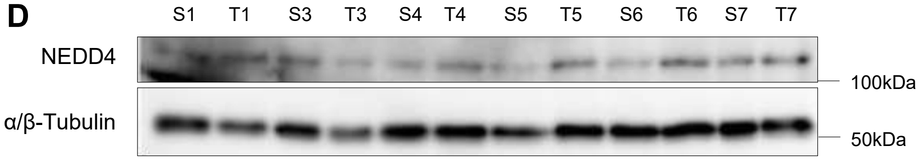

	S1	T1	S3	T3	S4	T4	S5	T5	S6	T6	S7	T7
NEDD4/ α/β-tubulin ratio	0.87	1.03	1.24	1.11	0.64	0.93	0.92	1.02	0.52	0.94	0.75	1.02

Figure 3. Images of Immunoblots (IB)

Gastrocnemius muscle (Fig.3A-D): .

A

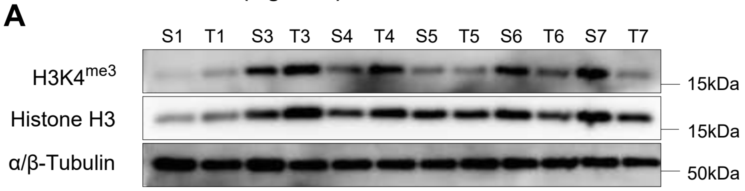

	S1	T1	S3	T3	S4	T4	S5	T5	S6	T6	S7	T7
H3K4me3 /Histone H3	0.25	0.46	0.75	0.78	0.45	0.44	0.30	0.21	0.43	0.43	0.50	0.31

B

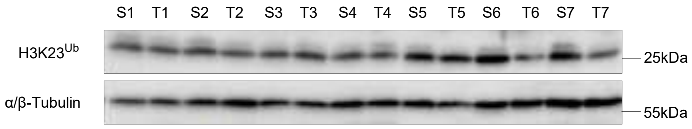

	S1	T1	S2	T2	S3	T3	S4	T4	S5	T5	S6	T6	S7	T7
H3K23Ub/ α/β-Tubulin Ratio	0.929	0.890	0.921	0.651	0.715	0.942	0.692	0.680	0.887	1.079	1.013	0.541	0.870	0.566

C

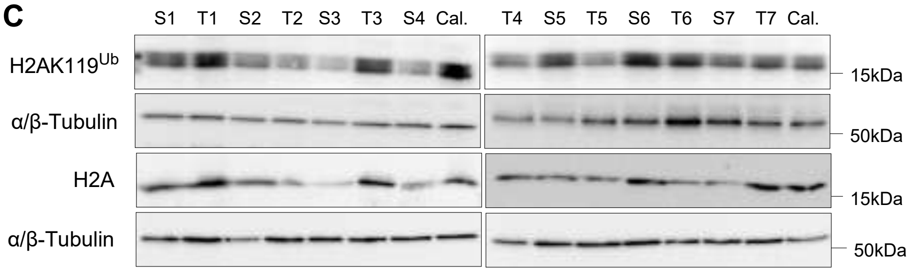

	S1	T1	S2	T2	S3	T3	S4	T4	S5	T5	S6	T6	S7	T7
H2AK119Ub/ α/β-Tubulin ratio	19.4	23.1	15.9	12.0	12.5	25.5	10.7	23.4	20.1	13.3	27.0	20.2	14.6	21.0
H2A/ α/β-Tubulin ratio	0.8	0.8	1.0	0.3	0.2	0.9	0.4	0.7	0.3	0.3	0.5	0.3	0.2	0.7
H2AK119Ub/ H2A ratio	24.0	29.5	16.0	50.6	75.9	29.0	28.1	68.2	64.5	24.7	77.9	91.1	20.4	16.7

Figure 3. Images of Immunoblots (IB)

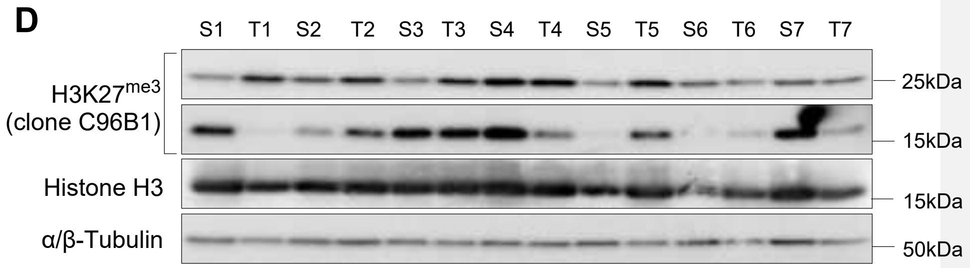

	S1	T1	S2	T2	S3	T3	S4	T4	S5	T5	S6	T6	S7
H3K27me3 (17kDa)/ Histone H3	0.73	0.19	0.26	0.65	1.39	1.53	2.34	0.48	0.13	0.84	0.08	0.24	0.97
H3K27me3 (25kDa)/ Histone H3	0.98	2.95	1.45	2.03	1.19	2.61	3.07	3.11	0.80	3.29	1.12	1.59	1.00

Figure 4A. Expression of EZH2 and MDM2 in plantaris, gastrocnemius and soleus muscles. All samples and identification of outliers confirmed by statistical analyses see method in main manuscript.

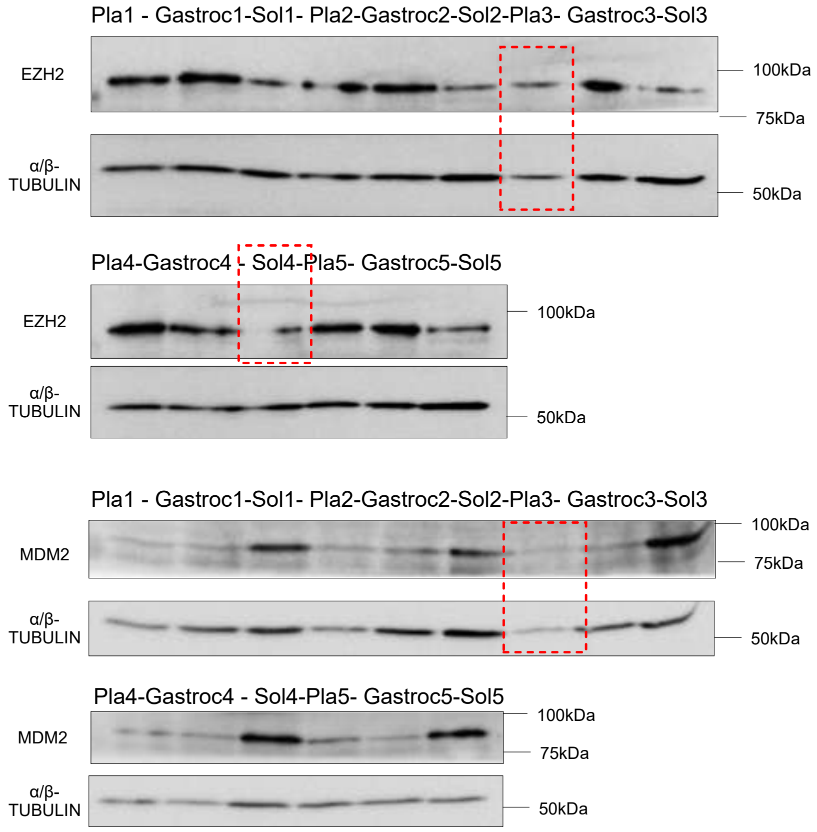

Animal ID	Pla1	Gastro c1	Sol1	Pla2	Gastro c2	Sol2	Pla3	Gastro c3	Sol3	Pla4	Gastro c4	Sol4	Pla5	Gastro c5	Sol5
EZH2 to α/β-TUBULIN	1.3	1.387	0.433	*1.022*	1.115	0.151	0.599	1.045	0.126	2.315	1.429	*0.316*	1.228	1.651	0.267
MDM2 (2A10) to α/β-TUBULIN	0.599	0.238	2.111	0.778	0.585	0.957	0.945	0.142	4.316	0.632	0.585	2.655	0.792	0.386	3.852

Figure 4B and C. Levels of EZH2 and MDM2 in plantaris and soleus after endurance training. All samples analysed and identification of outliers as per method see in main manuscript

Plantaris

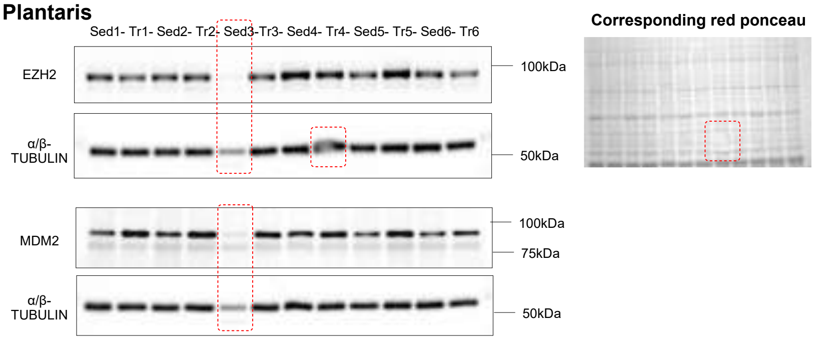

Animal ID	SED-1	TR-1	SED-2	TR-2	SED-3	TR-3	SED-4	TR-4	SED-5	TR-5	SED-6	TR-6
EZH2 to **α/β-TUBULIN**	0.553	0.410	0.565	0.551	0.014	0.498	0.862	0.576	0.518	0.864	0.469	0.379
MDM2 (2A10) to α/β-TUBULIN	0.145	0.518	0.197	0.470	0.008	0.342	0.187	0.383	0.153	0.449	0.132	0.223

NB: One outlier identified in red, confirmed statistically

Soleus

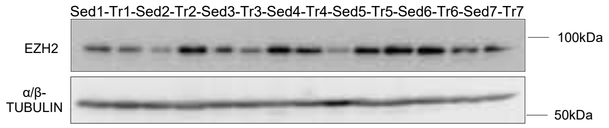

	SED1	TR1	SED2	TR2	SED3	TR3	SED4	TR4	SED5	TR5	SED6	TR6	SED7	TR7
**EZH2 to α/β-TUBULIN**	3.214	1.258	0.650	2.790	1.076	0.773	2.230	1.811	0.478	2.000	2.843	4.011	2.122	2.184

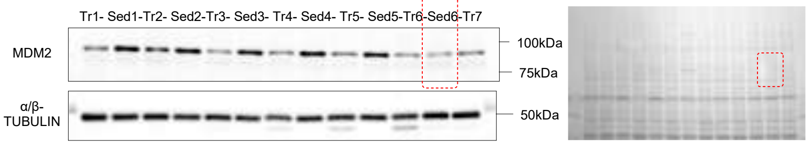

Animal ID	TR-1	SED-1	TR-2	SED-2	TR-3	SED-3	TR-4	SED-4	TR-5	SED-5	TR-6	SED-6	TR-7
MDM2 (2A10) to **α/β-tubulin**	0.230	0.789	0.412	0.834	0.206	0.768	0.313	0.906	0.312	0.764	0.265	0.134	0.311

Figure 5. Supplemental

Figure 5A. Comparison of H3K27me3 protein expression between muscles.

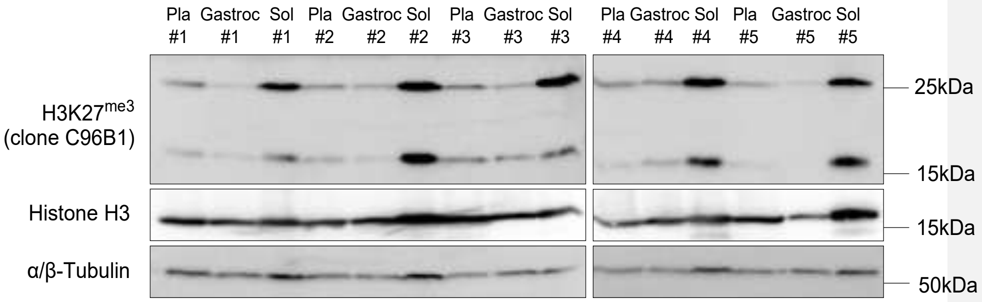

	Pla #1	Gastroc #1	Sol #1	Pla #2	Gastroc #2	Sol #2	Pla #3	Gastroc #3	Sol #3	Pla #4	Gastroc #4	Sol #4	Pla #5	Gastroc #5
H3K27me3 (25kDa)/ Histone H3	0.062	0.041	0.231	0.067	0.026	0.497	0.103	0.100	0.202	0.064	0.160	0.619	0.040	0.005
H3K27me3 (17kDa)/ Histone H3	0.138	0.050	0.667	0.131	0.081	0.648	0.110	0.076	1.198	0.336	0.245	1.196	0.130	0.066

Figure 5B. Expression of H3K27me3 in plantaris muscle.

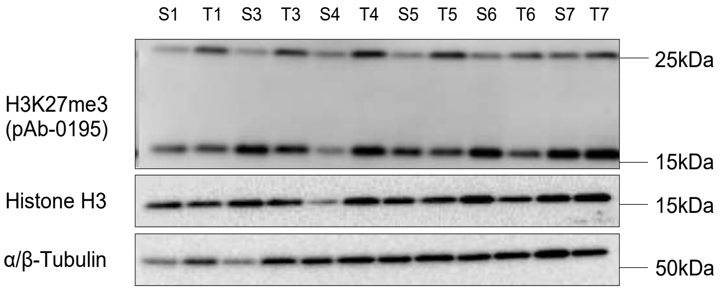

	S1	T1	S3	T3	S4	T4	S5	T5	S6	T6	S7	T7
**H3K27me3 (25kDa)/ Histone H3**	0.21	0.15	0.52	0.23	0.25	0.31	0.55	0.51	0.56	0.62	0.50	0.37
**H3K27me3 (25kDa)/ αβ-tubulin**	0.51	0.78	0.49	0.70	0.34	0.76	0.28	0.69	0.40	0.52	0.38	0.57
**H3K27me3 (17kDa)/ Histone H3**	0.59	0.64	0.80	0.73	0.74	0.79	0.61	0.62	0.69	0.66	0.79	0.80
**H3K27me3 (17kDa)/ αβ-tubulin**	1.43	0.91	2.59	0.99	0.49	1.09	0.76	0.68	1.11	0.69	0.98	1.24

Figure 5C. Expression of H3K27me3 in the soleus muscle.

NB: one outlier was identified and is indicated in red.

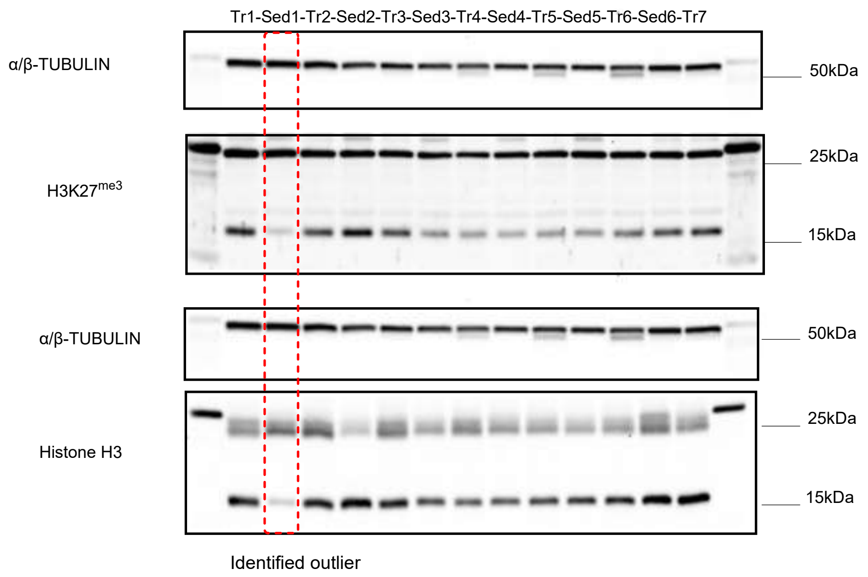

Animal ID	TR-1	*SED-1*	TR-2	SED-2	TR-3	SED-3	TR-4	SED-4	TR-5	SED-5	TR-6	SED-6	TR-7
H3K27me3 25 kDa to tubulin	0.936	*0.907*	0.904	1.171	1.122	0.928	0.886	0.946	1.153	1.191	1.450	1.078	0.828
H3K27me3 17 kDa to tubulin	0.912	*0.198*	0.967	1.591	1.235	0.656	0.680	0.486	0.748	0.591	1.144	1.011	1.007
H3 to tubulin	1.027	*4.579*	0.935	0.736	0.908	1.414	1.303	1.946	1.543	2.015	1.268	1.067	0.823
H3K27me3 25KDa to H3	0.912	*0.198*	0.967	1.591	1.235	0.656	0.680	0.486	0.748	0.591	1.144	1.011	1.007
H3K27me3 17KDa to H3	0.888	*0.043*	1.034	2.161	1.360	0.464	0.521	0.250	0.485	0.293	0.902	0.947	1.224

Figure 6. Images of Immunoblots (IB)

Figures 6A and 6B are representative immunoblots used to demonstrate the detection of the H3K27me3 protein at approximately 25kDa. The immunoblots are intended to show the reader that a protein band is detected in the muscle by several different antibodies (Figure 4A). In vitro, the detection of the H3K27me3 protein at 25kDa is more prominent in myotubes in comparison to endothelial cell (Figure 4B). These representative blots (panels) were included as visual representations only. Data analysis was not performed on these blots.

Figure 6C. replaces former figure 5A. DUB treated was originally just performed on control gastrocnemius muscle. See panel below. We have now repeated our experiment where we treated muscle extract with a cocktail of DUB (deubiquitylation enzymes). Treatment was performed on gastroscnemius protein extracts from sedentary and trained mice (see new figure 6C). This figure shows DUB treatement performed on 2 sedentary and 2 trained muscles. The detection of 25KDa band by the anti-H3K27me3 is more important in trained muscles and reduced by the DUB treatment. The anti-ubiquitin antibody is confirming the efficiency of the DUB treatment (see below and in new figure 6C). Figure 6C is qualitative analysis no densitometric analyses were performed.

Figure 6C replaces former figure 5A, see below. Mouse gastrocnemius protein samples were incubated with a control buffer (CON) or deubiquitylating buffer (DUB) for 1 hour prior to immunoblot.

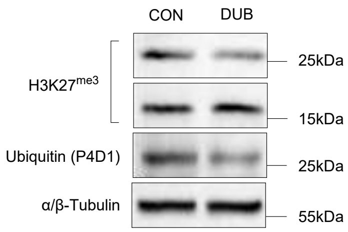

Fold Change	Control Buffer (CON)	Deubiquitinating Buffer (DUB)
Ubiquitin/ α/β-tubulin ratio	1.00	0.57
H3K27me3 (25kDa)/ α/β-tubulin ratio	1.00	0.55
H3K27me3 (17kDa)/ α/β-tubulin ratio	1.00	1.18

Figure 6D and E. H3K27^{me3} was immunoprecipitated from mice gastrocnemius muscle samples. Immunoblots were performed for ubiquitin and histone H3.

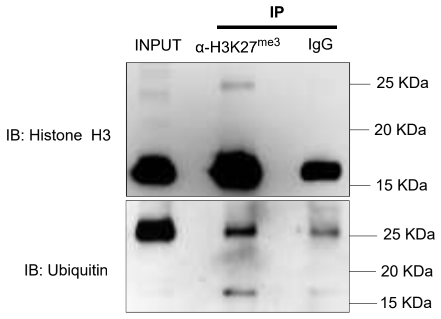

	IP to Input ratio	IgG to Input ratio	Fold of change: IP compared to IgG control
Ubiquitin	0.2658	0.1863	1.427
H3 25 kDA	0.3416	0	0.342
H3 17 kDA	1.5321	0.7635	2.007

Figures 6D and 6E are representative immunoblots used to demonstrate the detection of ubiquitin protein at approximately 25kDa following immunoprecipitation of protein samples with either a H3K27me3 (Fig.6D) or Histone H3 antibody (Fig.6E). This supports the notion that the detected band at 25kDa may be a ubiquitylated form of the histone. These representative blots (panels) were included as visual representations only. Data analysis was not performed on these blots.

Figure 6F. Representative raw chromatogram of the trypsin-digested 25 KDa band obtained after immuno-precipitation using a H3K27me3 antibody (gastrocnemius muscle protein extract).

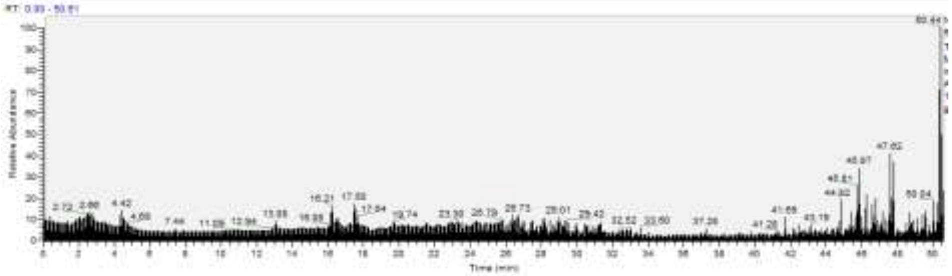

Figure 7. Supplemental

Figure 7A. mRNA expression in mice gastrocnemius muscles. Ct values (mean and SD per animal, qPCR performed in triplicate and average value was used) and fold of change *Vegfa, Thbs1, Kdr, Hhip, and Notch1*. *Hprt* was used as housekeeping gene.

Sample	*Hprt* Average CT	*Hprt* SD	*Vegfa* Average CT	*Vegfa* SD	*Vegfa* Δ Ct	*Vegfa* ΔΔ Ct	*Vegfa* Fold Change	*Thbs1* Average CT	*Thbs1* SD	*Thbs1* Δ Ct	*Thbs1* ΔΔ Ct	*Thbs1* Fold Change
Sedentary 1	25.41	0.03	26.31	0.10	0.90	-0.95	0.52	26.66	1.28	1.25	-1.09	0.47
Sedentary 2	26.88	0.08	26.79	0.20	-0.09	0.04	1.03	27.26	0.51	0.38	-0.22	0.86
Sedentary 3	26.52	0.07	26.69	0.29	0.17	-0.22	0.86	26.45	0.69	-0.07	0.22	1.17
Sedentary 4	25.80	0.22	26.41	0.24	0.62	-0.66	0.63	26.53	0.49	0.73	-0.57	0.67
Sedentary 5	26.23	0.08	25.91	0.16	-0.32	0.27	1.21	26.86	0.29	0.63	-0.47	0.72
Sedentary 6	26.11	0.19	25.29	0.77	-0.82	0.77	1.70	25.39	0.17	-0.72	0.88	1.84
Sedentary 7	26.12	0.06	25.32	0.13	-0.80	0.75	1.68	25.02	0.30	-1.10	1.26	2.40
Trained 1	26.55	0.10	26.30	0.18	-0.25	0.20	1.15	28.67	0.31	2.12	-1.96	0.26
Trained 2	26.80	0.06	26.12	0.10	-0.68	0.63	1.55	25.85	0.60	-0.95	1.11	2.16
Trained 3	26.49	0.32	25.24	0.02	-1.25	1.20	2.30	26.91	0.03	0.42	-0.26	0.84
Trained 4	26.02	0.13	25.04	0.60	-0.99	0.94	1.92	27.58	0.08	1.55	-1.40	0.38
Trained 5	26.17	0.13	26.19	0.14	0.01	-0.06	0.96	26.49	0.15	0.31	-0.15	0.90
Trained 6	26.18	0.24	25.07	0.71	-1.11	1.06	2.09	27.24	0.03	1.06	-0.90	0.53
Trained 7	25.91	0.20	25.13	0.05	-0.78	0.74	1.66	25.68	0.07	-0.23	0.39	1.31

Sample	*Hprt* Average CT	*Hprt* SD	*Kdr* Average CT	*Kdr* SD	*Kdr* Δ Ct	*Kdr* ΔΔ Ct	*Kdr* Fold Change	*Notch1* Average CT	*Notch1* SD	*Notch1* Δ Ct	*Notch1* ΔΔ Ct	*Notch1* Fold Change
Sedentary 1	27.13	0.17	28.37	0.02	1.24	-1.26	0.42	31.20	0.15	4.08	-0.29	0.82
Sedentary 2	28.71	0.29	28.69	0.06	-0.02	0.00	1.00	31.99	0.28	3.28	0.50	1.42
Sedentary 3	29.08	0.33	28.84	0.20	-0.24	0.22	1.16	32.81	0.40	3.74	0.05	1.03
Sedentary 4	27.56	0.44	28.01	0.17	0.44	-0.47	0.72	31.91	0.51	4.48	-0.69	0.62
Sedentary 5	27.67	0.44	27.11	0.18	-0.57	0.54	1.46	31.78	0.19	4.11	-0.32	0.80
Sedentary 6	29.25	0.17	29.05	0.09	-0.19	0.17	1.12	32.96	0.39	3.72	0.06	1.05
Sedentary 7	28.16	0.13	27.33	0.05	-0.83	0.80	1.75	31.24	0.08	3.08	0.70	1.63
Trained 1	27.49	0.26	28.70	0.45	1.21	-1.24	0.42	30.84	0.43	3.35	0.43	1.35
Trained 2	27.87	0.37	28.19	0.47	0.32	-0.35	0.79	30.37	0.28	2.50	1.28	2.43
Trained 3	27.67	0.38	28.04	0.10	0.37	-0.40	0.76	30.47	0.30	2.80	0.98	1.97
Trained 4	27.87	0.39	28.34	0.11	0.47	-0.49	0.71	31.13	0.12	3.45	0.33	1.26
Trained 5	27.53	0.28	29.06	0.07	1.53	-1.55	0.34	31.34	0.21	3.81	-0.03	0.98
Trained 6	27.91	0.48	28.56	0.20	0.65	-0.67	0.63	31.07	0.03	3.16	0.62	1.54
Trained 7	27.70	0.75	28.32	0.13	0.62	-0.64	0.64	30.74	0.34	3.05	0.74	1.66

Sample	*Hprt* Average CT	*Hprt* SD	*Hhip* Average CT	*Hhip* SD	*Hhip* Δ Ct	*Hhip* ΔΔ Ct	*Hhip* Fold Change
Sedentary 1	27.13	0.17	34.26	1.00	7.14	-2.91	0.13
Sedentary 2	28.71	0.29	33.23	0.28	4.52	-0.29	0.82
Sedentary 3	29.08	0.33	31.85	0.52	2.77	1.45	2.73
Sedentary 4	27.56	0.44	32.81	0.54	5.38	-1.16	0.45
Sedentary 5	27.67	0.44	32.18	0.04	4.51	-0.28	0.82
Sedentary 6	29.25	0.17	32.32	0.10	3.07	1.15	2.22
Sedentary 7	28.16	0.13	30.34	0.32	2.18	2.04	4.12
Trained 1	27.49	0.26	32.25	0.52	4.76	-0.54	0.69
Trained 2	27.87	0.37	32.54	0.44	4.68	-0.45	0.73
Trained 3	27.67	0.38	33.20	0.45	5.54	-1.31	0.40
Trained 4	27.87	0.39	33.60	0.41	5.92	-1.69	0.31
Trained 5	27.53	0.28	33.54	0.07	6.01	-1.78	0.29
Trained 6	27.91	0.48	34.32	0.45	6.41	-2.19	0.22
Trained 7	27.70	0.75	31.44	0.23	3.74	0.48	1.40

Figure 7B. Chromatin extracts from gastrocnemius muscles were subjected to a targeted ChIP assay using an anti-H3K27^{me3} antibody (pAb195) and using Taqman probes for *Kdr* and *Notch1*. Grubb's test (α=0.05) for DNA quantity of INPUT was used to detect outliers. The "Trained 2" ChIP samples was identified as an outlier and was excluded from analysis.

Commented [ER1]: Should we show this table first and then the Ct table without the Trained 2?

Gastrocnemius muscle	DNA content
Sedentary 3	3.86725
Sedentary 4	1.840625
Sedentary 5	2.772875
Sedentary 7	1.169625
Trained 1	0.751625
Trained 2	4.44425
Trained 3	0.67375
Trained 4	0.32425

Ct of valued obtained using *Kdr* TSS probe.

Gastrocnemius muscle	Ct of INPUT	Ct of α-H3K27me3 ChIP product	Fold of change compared to INPUT
Sedentary 3	30.15255	28.85405	0.122987
Sedentary 4	31.24883	29.25424	0.199252
Sedentary 5	30.11232	28.05069	0.208729
Sedentary 7	32.1543	28.33418	0.706218
Trained 1	32.98011	28.69078	0.977657
Trained 2	29.13718	28.60399	0.072357
Trained 3	31.47492	26.6305	1.436427
Trained 4	33.40828	29.48368	0.759266

Ct of valued obtained using *Notch1* TSS probe.

Gastrocnemius muscle	Ct of INPUT	Ct of α-H3K27me3 ChIP product	Fold of change compared to INPUT
Sedentary 3	29.4367	27.66	0.171982
Sedentary 4	30.8200	28.22	0.303408
Sedentary 5	29.6893	27.10	0.302079
Sedentary 7	31.1630	27.82	0.509446
Trained 1	32.4237	28.03	1.050202
Trained 2	28.2720	27.56	0.082203
Trained 3	30.6543	25.95	1.304352
Trained 4	32.8917	29.23	0.634489

Figure 7C to E Chromatin extracts from gastrocnemius muscles were subjected to a targeted ChIP assay using an anti-$H3K27^{me3}$ antibody (pAb195) and using Taqman probes for *Kdr* and *Notch1*. Grubb's test (α=0.05) for DNA quantity of INPUT was used to detect outliers. No outliers were identified.

Sample	DNA quantity of input (ng/µl)
Gastrocnemius-1	10.08
Gastrocnemius-2	7.75
Gastrocnemius-3	9.79
Gastrocnemius-4	12.38
Gastrocnemius-5	17.04
Gastrocnemius-6	6.54
Gastrocnemius-7	10.8
Gastrocnemius-8	12.5

Figure 7C.

Ct values and % of INPUT for KDR TSS regions

Sample	KDR	KDR'	KDR Average
Gastrocnemius-1 INPUT (1:20) C_t	31.077	30.920	
Gastrocnemius-1 $H3K27^{me3}$ C_t	33.337	32.476	
Gastrocnemius-1 $H3K27^{me3}$ **% of INPUT**	0.174	0.283	0.229
Gastrocnemius-2 INPUT (1:20) C_t	32.152	31.870	
Gastrocnemius-2 $H3K27^{me3}$ C_t	31.864	31.841	
Gastrocnemius-2 $H3K27^{me3}$ **% of INPUT**	1.017	0.851	0.934
Gastrocnemius-3 INPUT (1:20) C_t	31.648	31.601	
Gastrocnemius-3 $H3K27^{me3}$ C_t	32.523	31.608	
Gastrocnemius-3 $H3K27^{me3}$ **% of INPUT**	0.454	0.829	0.641

Gastrocnemius-4 INPUT (1:20) ***C_t***	30.000	29.398	
Gastrocnemius-4 H3K27^{me3} ***C_t***	32.470	32.172	
Gastrocnemius-4 H3K27^{me3} **% of INPUT**	0.150	0.122	0.136
Gastrocnemius-5 INPUT (1:2) ***C_t***	26.715	26.747	
Gastrocnemius-5 H3K27^{me3} ***C_t***	30.029	30.154	
Gastrocnemius-5 H3K27^{me3} **% of INPUT**	0.838	0.785	0.811
Gastrocnemius-6 INPUT (1:2) ***C_t***	30.422	30.416	
Gastrocnemius-6 H3K27^{me3} ***C_t***	31.857	31.959	
Gastrocnemius-6 H3K27^{me3} **% of INPUT**	0.465	0.429	0.446
Gastrocnemius-7 INPUT (1:2) ***C_t***	29.576	29.502	
Gastrocnemius-7 H3K27^{me3} ***C_t***	31.438	31.373	
Gastrocnemius-7 H3K27^{me3} **% of INPUT**	0.344	0.342	0.343
Gastrocnemius-8 INPUT (1:2) ***C_t***	29.463	29.382	
Gastrocnemius-8 H3K27^{me3} ***C_t***	31.911	31.926	
Gastrocnemius-8 H3K27^{me3} **% of INPUT**	0.229	0.214	0.222
Gastrocnemius-1 INPUT (1:20) ***C_t***	31.077	30.920	
Gastrocnemius-1 H3K4^{me3} ***C_t***	31.959	32.014	
Gastrocnemius-1 H3K4^{me3} **% of INPUT**	0.452	0.390	0.421
Gastrocnemius-2 INPUT (1:20) ***C_t***	32.152	31.870	
Gastrocnemius-2 H3K4^{me3} ***C_t***	31.430	31.185	
Gastrocnemius-2 H3K4^{me3} **% of INPUT**	1.375	1.340	1.357
Gastrocnemius-3 INPUT (1:20) ***C_t***	32.483	32.067	
Gastrocnemius-3 H3K4^{me3} ***C_t***	31.648	31.601	
Gastrocnemius-3 H3K4^{me3} **% of INPUT**	0.467	0.603	0.535
Gastrocnemius-4 INPUT (1:20) ***C_t***	30.000	29.398	
Gastrocnemius-4 H3K4^{me3} ***C_t***	34.509	34.202	
Gastrocnemius-4 H3K4^{me3} **% of INPUT**	0.037	0.030	0.033

Gastrocnemius-5 INPUT (1:20) C_t	26.715	26.747	
Gastrocnemius-5 H3K4^{me3} C_t	32.218	31.973	
Gastrocnemius-5 H3K4^{me3} **% of INPUT**	0.184	0.223	0.203
Gastrocnemius-6 INPUT (1:20) C_t	30.422	30.416	
Gastrocnemius-6 H3K4^{me3} C_t	33.783	33.286	
Gastrocnemius-6 H3K4^{me3} **% of INPUT**	0.122	0.171	0.146
Gastrocnemius-7 INPUT (1:20) C_t	29.576	29.502	
Gastrocnemius-7 H3K4^{me3} C_t	32.711	32.799	
Gastrocnemius-7 H3K4^{me3} **% of INPUT**	0.142	0.127	0.135
Gastrocnemius-8 INPUT (1:20) C_t	29.463	29.382	
Gastrocnemius-8 H3K4^{me3} C_t	33.300	33.501	
Gastrocnemius-8 H3K4^{me3} **% of INPUT**	0.087	0.072	0.080

Ct values and % of INPUT for Notch1 TSS regions

**Sample**	**Notch1**	**Notch1'**	**Notch1"**	**KDR Average**
Gastrocnemius-1 INPUT (1:20) C_t	31.395	30.483	31.406	
Gastrocnemius-1 H3K27^{me3} C_t	32.040	32.773	32.318	
Gastrocnemius-1 H3K27^{me3} **% of INPUT**	0.533	0.170	0.443	0.382
Gastrocnemius-2 INPUT (1:20) C_t	32.681	32.037	32.374	
Gastrocnemius-2 H3K27^{me3} C_t	31.334	30.741	31.411	
Gastrocnemius-2 H3K27^{me3} **% of INPUT**	2.12	2.046	1.624	0.193
Gastrocnemius-3 INPUT (1:20) C_t	31.501	31.281	31.617	
Gastrocnemius-3 H3K27^{me3} C_t	31.658	31.457	31.530	
Gastrocnemius-3 H3K27^{me3} **% of INPUT**	0.747	0.737	0.885	0.790
Gastrocnemius-4 INPUT (1:20) C_t	29.842	29.168	29.823	
Gastrocnemius-4 H3K27^{me3} C_t	31.811	30.247	31.653	
Gastrocnemius-4 H3K27^{me3} **% of INPUT**	0.178	0.157	0.234	0.190
Gastrocnemius-5 INPUT (1:2) C_t	26.644	25.395	26.594	

Gastrocnemius-5 H3K27^{me3} C_t	29.901	28.751	29.857	
Gastrocnemius-5 H3K27^{me3} **% of INPUT**	0.872	0.813	0.868	0.851
Gastrocnemius-6 INPUT (1:2) C_t	30.711	30.013	30.336	
Gastrocnemius-6 H3K27^{me3} C_t	31.383	30.827	31.117	
Gastrocnemius-6 H3K27^{me3} **% of INPUT**	0.784	0.711	0.728	0.741
Gastrocnemius-7 INPUT (1:2) C_t	29.648	29.034	29.422	
Gastrocnemius-7 H3K27^{me3} C_t	30.920	30.380	30.640	
Gastrocnemius-7 H3K27^{me3} **% of INPUT**	0.517	0.532	0.546	0.532
Gastrocnemius-8 INPUT (1:2) C_t	29.658	29.034	29.422	
Gastrocnemius-8 H3K27^{me3} C_t	31.430	30.840	30.876	
Gastrocnemius-8 H3K27^{me3} **% of INPUT**	0.366	0.357	0.456	0.393
Gastrocnemius-1 INPUT (1:20) C_t	31.395	30.483	31.406	
Gastrocnemius-1 H3K4^{me3} C_t	31.886	30.962	31.937	
Gastrocnemius-1 H3K4^{me3} **% of INPUT**	0.591	0.598	0.577	0.589
Gastrocnemius-2 INPUT (1:20) C_t	32.681	32.037	32.374	
Gastrocnemius-2 H3K4^{me3} C_t	31.123	30.862	31.251	
Gastrocnemius-2 H3K4^{me3} **% of INPUT**	2.454	1.881	1.814	2.050
Gastrocnemius-3 INPUT (1:20) C_t	31.501	31.281	31.617	
Gastrocnemius-3 H3K4^{me3} C_t	32.325	32.510	32.544	
Gastrocnemius-3 H3K4^{me3} **% of INPUT**	0.471	0.355	0.438	0.421
Gastrocnemius-4 INPUT (1:20) C_t	29.842	29.168	29.823	
Gastrocnemius-4 H3K4^{me3} C_t	33.059	32.551	33.302	
Gastrocnemius-4 H3K4^{me3} **% of INPUT**	0.090	0.080	0.075	0.081
Gastrocnemius-5 INPUT (1:20) C_t	26.644	25.395	26.594	
Gastrocnemius-5 H3K4^{me3} C_t	31.811	30.247	31.623	
Gastrocnemius-5 H3K4^{me3} **% of INPUT**	0.232	0.289	0.255	0.259

Gastrocnemius-6 INPUT (1:20) C_t	30.711	30.013	30.336	
Gastrocnemius-6 H3K4^{me3} C_t	32.827	32.150	32.726	
Gastrocnemius-6 H3K4^{me3} **% of INPUT**	0.288	0.284	0.239	0.270
Gastrocnemius-7 INPUT (1:20) C_t	29.648	29.034	29.422	
Gastrocnemius-7 H3K4^{me3} C_t	32.369	31.583	32.136	
Gastrocnemius-7 H3K4^{me3} **% of INPUT**	0.190	0.231	0.193	0.205
Gastrocnemius-8 INPUT (1:20) C_t	29.658	29.034	29.422	
Gastrocnemius-8 H3K4^{me3} C_t	32.741	31.910	32.488	
Gastrocnemius-8 H3K4^{me3} **% of INPUT**	0.147	0.170	0.149	0.156

Figure 7D

***Notch1* TSS (Correlation)**

Sample	H3K27^{me3} (% of INPUT)	H3K4^{me3} (% of INPUT)
Gastrocnemius-1	0.382	0.589
Gastrocnemius-2	1.930	2.050
Gastrocnemius-3	0.790	0.421
Gastrocnemius-4	0.190	0.081
Gastrocnemius-5	0.851	0.259
Gastrocnemius-6	0.741	0.270
Gastrocnemius-7	0.532	0.205
Gastrocnemius-8	0.393	0.156

Figure 7E

***Kdr* TSS (Correlation)**

Sample	H3K27^{me3} (% of INPUT)	H3K4^{me3} (% of INPUT)
Gastrocnemius-1	0.229	0.421
Gastrocnemius-2	0.934	1.357
Gastrocnemius-3	0.641	0.535
Gastrocnemius-4	0.136	0.033
Gastrocnemius-5	0.811	0.203
Gastrocnemius-6	0.446	0.146
Gastrocnemius-7	0.343	0.135
Gastrocnemius-8	0.222	0.080

Figure 7F

Sample	KDR	NOTCH-1'
(Tibialis Anterior) Sedentary-3 INPUT (1:10) ***C_t***	29.426	28.169
(Tibialis Anterior) Sedentary-3 H3K9^{Ac} ***C_t***	37.708	33.297
(Tibialis Anterior) Sedentary-3 H3K9^{Ac} **% of INPUT**	0.005355523	0.047657647
(Tibialis Anterior) Sedentary-4 INPUT (1:10) ***C_t***	28.358	27.106
(Tibialis Anterior) Sedentary-4 H3K9^{Ac} ***C_t***	35.058	32.855
(Tibialis Anterior) Sedentary-4 H3K9^{Ac} **% of INPUT**	0.016026442	0.030997199
(Tibialis Anterior) Sedentary-5 INPUT (1:10) ***C_t***	28.506	27.431
(Tibialis Anterior) Sedentary-5 H3K9^{Ac} ***C_t***	34.592	32.767
(Tibialis Anterior) Sedentary-5 H3K9^{Ac} **% of INPUT**	0.024545367	0.041273765
(Tibialis Anterior) Sedentary-7 INPUT (1:10) ***C_t***	28.589	27.423
(Tibialis Anterior) Sedentary-7 H3K9^{Ac} ***C_t***	32.911	30.556
(Tibialis Anterior) Sedentary-7 H3K9^{Ac} **% of INPUT**	0.083373817	0.190073498
(Tibialis Anterior) Trained-1 INPUT (1:10) ***C_t***	28.163	27.039
(Tibialis Anterior) Trained-1 H3K9^{Ac} ***C_t***	33.595	31.465
(Tibialis Anterior) Trained-1 H3K9^{Ac} **% of INPUT**	0.038592257	0.077533883
(Tibialis Anterior) Trained-2 INPUT (1:10) ***C_t***	28.463	27.281
(Tibialis Anterior) Trained-2 H3K9^{Ac} ***C_t***	35.031	33.235
(Tibialis Anterior) Trained-2 H3K9^{Ac} **% of INPUT**	0.017575784	0.026890026
(Tibialis Anterior) Trained-3 INPUT (1:10) ***C_t***	28.564	27.395
(Tibialis Anterior) Trained-3 H3K9^{Ac} ***C_t***	34.372	32.247
(Tibialis Anterior) Trained-3 H3K9^{Ac} **% of INPUT**	0.029760613	0.05769751
(Tibialis Anterior) Trained-4 INPUT (1:10) ***C_t***	30.242	29.029
(Tibialis Anterior) Trained-4 H3K9^{Ac} ***C_t***	37.331	36.072
(Tibialis Anterior) Trained-4 H3K9^{Ac} **% of INPUT**	0.012234845	0.012642453

Figure 7G

Sample	KDR<br>H3K9^{Ac} (% of INPUT)	NOTCH-1'<br>H3K9^{Ac} (% of INPUT)
(Tibialis Anterior) Trained-1	0.038592257	0.077533883
(Tibialis Anterior) Trained-2	0.017575784	0.026890026
(Tibialis Anterior) Trained-3	0.029760613	0.05769751
(Tibialis Anterior) Trained-4	0.012234845	0.012642453

Figure 8 Expression of *Notch1* and *Kdr* in soleus muscle from sedentary and trained mice.

mRNA level of *Kdr* and *Notch1* in the soleus muscles of sedentary and trained mice

Sample ID	*Hprt* Average Ct	*Hprt* SD	*Notch1* Average CT	*Notch1* SD	*Notch1* ΔCt	*Notch1* ΔΔCt	*Notch1* Fold Change	*Kdr* Average CT	*Kdr* SD	*Kdr* ΔCt	*Kdr* ΔΔCt	*Kdr* Fold Change
**Sedentary 1**	26.85	0.13	29.00	0.016	2.14	0.37	0.78	25.51	0.07	-1.34	0.09	0.94
**Sedentary 2**	27.21	0.12	29.16	0.044	1.95	0.17	0.89	25.72	0.01	-1.49	-0.06	1.04
**Sedentary 3**	27.48	0.05	28.92	0.025	1.44	-0.34	1.27	25.76	0.03	-1.73	-0.29	1.23
**Sedentary 4**	27.42	0.06	28.84	0.006	1.43	-0.35	1.27	25.57	0.03	-1.84	-0.41	1.33
**Sedentary 5**	27.47	0.05	29.02	0.049	1.55	-0.23	1.17	26.21	0.08	-1.26	0.17	0.89
**Sedentary 6**	27.32	0.06	29.29	0.102	1.98	0.20	0.87	26.07	0.06	-1.25	0.18	0.88
**Sedentary 7**	27.39	0.10	29.20	0.060	1.82	0.04	0.97	26.13	0.04	-1.25	0.18	0.88
**Trained 1**	27.10	0.04	29.42	0.035	2.32	0.54	0.69	25.75	0.02	-1.35	0.08	0.95
**Trained 2**	27.18	0.08	29.55	0.050	2.37	0.59	0.67	25.92	0.01	-1.26	0.17	0.89
**Trained 3**	27.25	0.30	29.35	0.009	2.10	0.32	0.80	25.46	0.03	-1.79	-0.36	1.28
**Trained 4**	27.10	0.14	29.32	0.090	2.22	0.44	0.74	25.56	0.02	-1.54	-0.11	1.08
**Trained 5**	27.11	0.04	29.02	0.028	1.91	0.14	0.91	25.71	0.03	-1.40	0.03	0.98
**Trained 6**	27.42	0.05	29.31	0.172	1.89	0.11	0.93	26.17	0.01	-1.26	0.17	0.89
**Trained 7**	27.08	0.09	29.26	0.020	2.18	0.41	0.75	25.90	0.05	-1.18	0.25	0.84

Figure 9 Western blot images

Figure 9A. Immunoblots of differentiating C2C12 myoblasts. The original blot with table of data is included below. A representative blot was redone for illustrative purposes. **UD =** undifferentiated, **2D =** 2 day differentiated, **4D =** 4 day differentiated, **7D =** 7 day differentiated. A calibrator consisting of pooled protein samples ("POOL") was included to standardize samples between different membranes.

Representative Blot Image

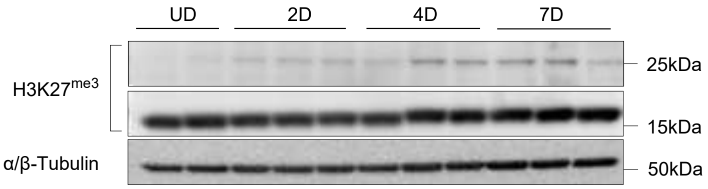

Original Blot (data analyzed)

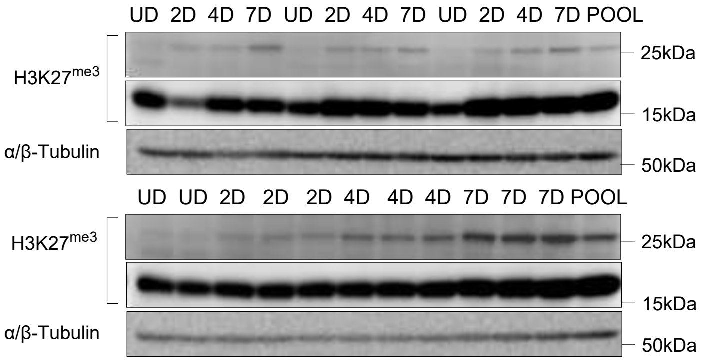

	UD #1	2D #1	4D #1	7D #1	UD #2	2D #2	4D #2	7D #2	UD #3	2D #3	4D #3	7D #3
H3K27me3 (25kDa)/ α/β-Tubulin ratio	0.081	0.257	0.488	1.186	0.197	0.380	0.497	0.987	0.232	0.304	0.789	1.019
H3K27me3 (17kDa)/ α/β-Tubulin ratio	2.145	2.320	1.614	1.302	2.342	2.743	1.480	1.095	1.319	3.207	1.447	0.974

	UD #4	UD #5	2D #4	2D #5	2D #6	4D #4	4D #5	4D #6	7D #4	7D #5	7D #6
H3K27me3 (25kDa)/ α/β-Tubulin ratio	0.259	0.248	0.259	0.236	0.235	0.398	0.388	0.352	0.547	0.482	0.623
H3K27me3 (17kDa)/ α/β-Tubulin ratio	1.377	1.138	1.278	1.530	1.216	1.245	1.448	1.308	1.317	1.352	1.392

Figure 9B - Western blotting images

n=6 biological replicate of C2C12 myotubes treated with MG132 (M#) or control vehicle (DMSO,D#)

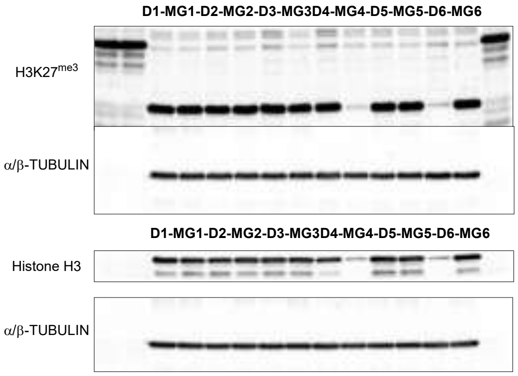

Corresponding Ponceau

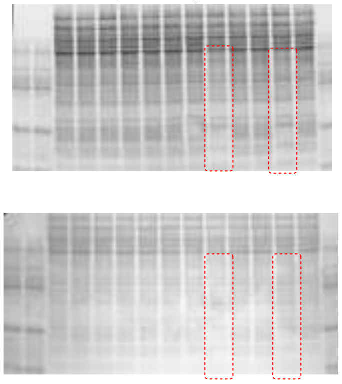

Identified outliers are MG132-4 and DMSO-6

C2C12 biological replicate n=6	DMSO-1	MG132-1	DMSO-2	MG132-2	DMSO-3	MG132-3	DMSO-4	MG132-4	DMSO-5	MG132-5	DMSO-6	MG132-6
**H3K27me3 25 kDa to α/β-TUBULIN**	0.174	0.207	0.351	0.275	0.471	0.190	0.557	**0.341**	0.334	0.266	**0.429**	0.310
**H3K27me3 17 kDa to α/β-TUBULIN**	0.967	1.032	0.961	1.130	1.224	0.953	0.946	**0.006**	1.258	1.166	**0.008**	1.082
**Histone H3 to to α/β-TUBULIN**	1.170	0.984	1.010	1.092	1.038	1.006	0.959	**0.155**	1.374	1.120	**0.160**	1.378
**H3K27me3 25 KDA to H3**	0.149	0.210	0.347	0.2521	0.454	0.1887	0.581	**2.196**	0.243	0.2379	**2.684**	0.2253
**H3K27me3 17KDa to H3**	0.826	1.0489	0.951	1.0344	1.179	0.9478	1.179	**0.9478**	0.916	1.0411	**0.049**	0.7856

Figure 10. Supplemental

Figure 10A. C2C12 myotubes were incubated with 1µM or 5µM of GSK343 (E inhibitor) for 48h.

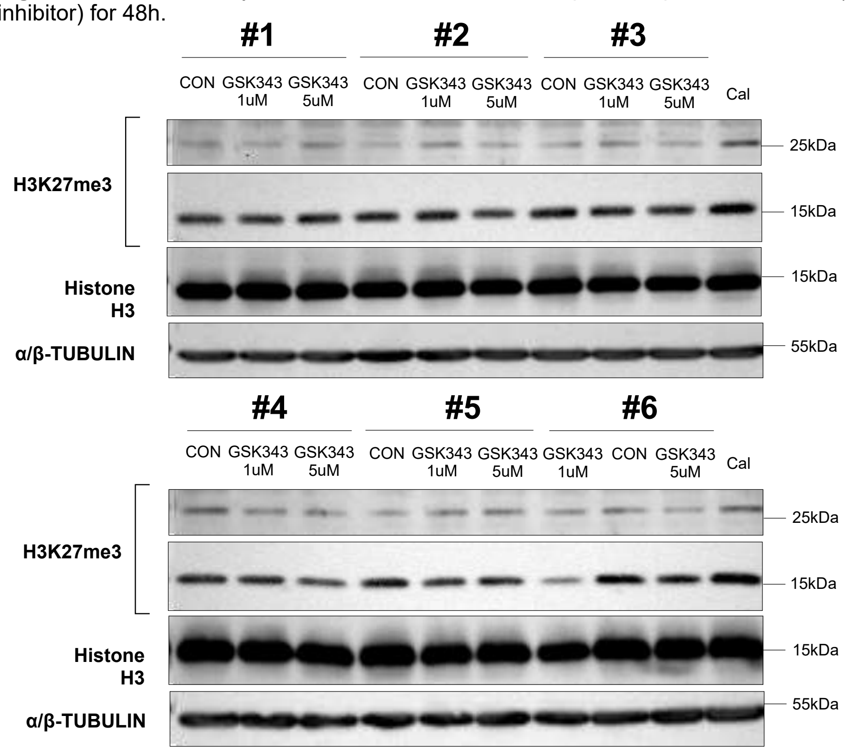

	CON #1	1uM GSK #1	5uM GSK #1	CON #2	1uM GSK #2	5uM GSK #2	CON #3	1uM GSK #3	5uM GSK #3
H3K27me3 (25kDa)/ Histone H3	0.59	0.51	0.87	0.53	0.99	0.71	0.77	1.03	0.69
H3K27me3 (17kDa)/ Histone H3	0.82	0.64	0.74	0.80	0.81	0.64	0.96	0.84	0.73

	CON #4	1uM GSK #4	5uM GSK #4	CON #5	1uM GSK #5	5uM GSK #5	1uM GSK #6	CON #6	5uM GSK #6
H3K27me3 (25kDa)/ Histone H3	1.22	0.81	0.81	0.59	1.16	0.98	0.77	0.90	0.64
H3K27me3 (17kDa)/ Histone H3	0.92	0.75	0.59	0.90	0.82	0.73	0.30	0.93	0.75

Figure 10. Supplemental

Figure 10 C2C12 myotubes were treated with GSK343 for 48h. Myotubes were subsequently subjected to electric pulse stimulation (EPS), 90 min per day for 4 consecutive days.

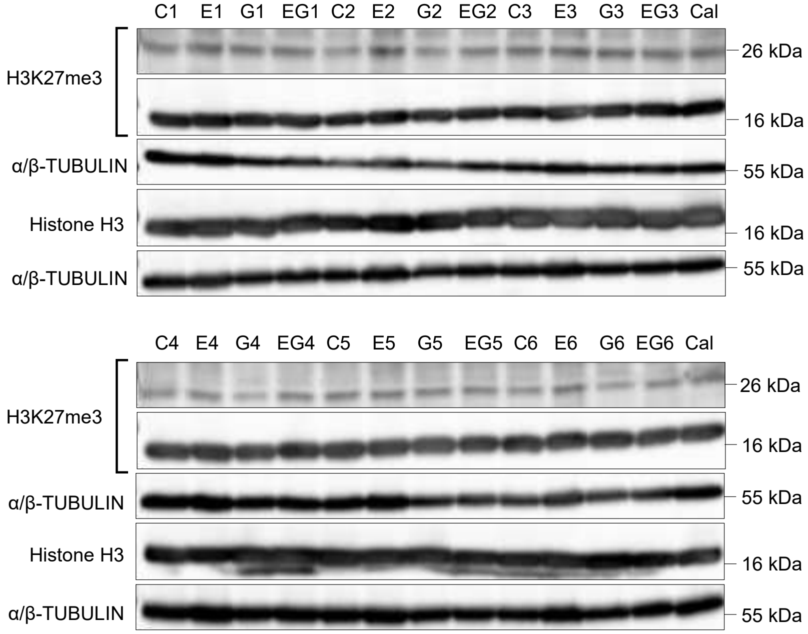

	C1	E1	G1	EG1	C2	E2	G2	EG2	C3	E3	G3	EG3
H3K27me3 (25kDa)/ Histone H3	1.23	1.35	1.17	1.27	1.14	1.59	1.14	1.16	1.17	1.75	1.17	1.35
H3K27me3 (17kDa)/ Histone H3	1.03	1.06	0.97	1.19	1.21	1.14	0.95	0.98	1.06	1.54	1.02	1.27

	C4	E4	G4	EG4	C5	E5	G5	EG5	C6	E6	G6	EG6
H3K27me3 (25kDa)/ Histone H3	1.06	1.55	0.87	1.35	1.33	1.59	1.60	1.55	0.93	2.13	0.83	1.02
H3K27me3 (17kDa)/ Histone H3	0.99	1.06	0.81	1.08	1.11	1.05	1.12	1.37	1.29	1.59	0.99	1.12

Figure 10. Supplemental

Figure 10C. *Notch1* mRNA. C2C12 myotubes were treated with GSK343 for 48h.

Sample ID	*Notch1* Average CT	*Notch1* SD	*Hprt* Average CT	*Hprt* SD	Δ Ct sample	ΔΔ Ct	Fold increase
CON #1	28.84	0.04	25.35	0.11	3.49	0.16	1.12
CON #2	28.48	0.04	24.67	0.09	3.80	-0.16	0.90
CON #3	28.29	0.09	24.63	0.08	3.65	-0.01	1.00
CON #4	28.19	0.05	25.66	0.14	2.53	-0.16	0.90
CON #5	27.51	0.08	25.33	0.09	2.17	0.20	1.15
CON #6	27.80	0.05	25.39	0.19	2.41	-0.04	0.97
GSK #1	27.78	0.15	25.31	0.13	2.47	1.18	2.26
GSK #2	27.85	0.12	25.19	0.12	2.66	0.99	1.99
GSK #3	27.42	0.14	24.85	0.11	2.57	1.07	2.11
GSK#4	27.20	0.11	25.53	0.16	1.67	0.70	1.63
GSK#5	27.64	0.05	25.91	0.05	1.73	0.64	1.56
GSK#6	27.68	0.03	26.17	0.10	1.51	0.86	1.82

Figure 10D. *Notch1* mRNA. C2C12 myotubes were treated with GSK343 for 48 hours and then with EPS (90 minutes per day for 4 days in the presence of GSK343).

Sample ID	Average CT Notch1	Notch1 SD	Average CT Hprt	Hprt SD	Δ Ct sample	ΔΔ Ct	Fold increase
DMSO #1	28.21	0.10	24.86	0.07	3.35	0.39	1.31
DMSO #2	28.11	0.03	24.25	0.08	3.86	-0.13	0.92
DMSO #3	28.64	0.07	24.67	0.03	3.96	-0.23	0.85
DMSO #4	28.40	0.05	24.63	0.05	3.77	-0.03	0.98
DMSO + EPS #1	26.10	0.06	23.69	0.03	2.41	1.33	2.51
DMSO + EPS #2	26.17	0.01	24.38	0.12	1.79	1.95	3.85
DMSO + EPS #3	26.45	0.04	23.89	0.24	2.56	1.17	2.26
DMSO + EPS #4	25.99	0.00	24.06	0.07	1.92	1.81	3.52
GSK343 #1	27.34	0.20	24.62	0.04	2.72	1.02	2.03
GSK343 #2	27.85	0.10	25.16	0.05	2.69	1.04	2.06
GSK343 #3	27.76	0.01	23.99	0.01	3.78	-0.04	0.97
GSK343 #4	27.82	0.05	24.57	0.05	3.25	0.49	1.40
GSK343 + EPS #1	27.02	0.04	24.54	0.08	2.48	1.26	2.40
GSK343 + EPS #2	26.97	0.19	24.14	0.30	2.83	0.91	1.87
GSK343 + EPS #3	26.68	0.09	23.62	0.06	3.06	0.68	1.60
GSK343 + EPS #4	26.91	0.08	23.81	0.06	3.10	0.63	1.55

Figure 11. Supplemental

Figure 11A. C2C12 myotubes were differentiated for 1-5 days.

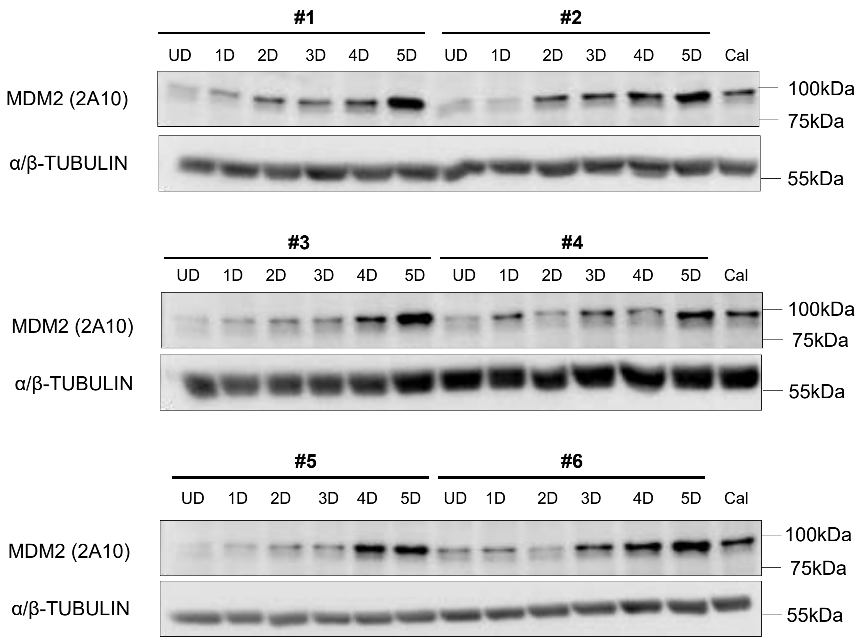

	UD #1	1D #1	2D #1	3D #1	4D #1	5D #1	UD #2	1D #2	2D #2	3D #2	4D #2	5D #2
MDM2/ α/β-TUBULIN	0.452	0.513	0.906	0.712	1.09	2.923	0.356	0.285	0.872	0.928	1.237	2.49

	UD #3	1D #3	2D #3	3D #3	4D #3	5D #3	UD #4	1D #4	2D #4	3D #4	4D #4	5D #4
MDM2/ α/β-TUBULIN	0.398	0.769	1.084	0.959	1.703	3.05	0.543	0.632	1.024	0.984	0.852	1.71

	UD #5	1D #5	2D #5	3D #5	4D #5	5D #5	UD #6	1D #6	2D #6	3D #6	4D #6	5D #6
MDM2/ α/β-TUBULIN	0.205	0.388	0.666	0.653	1.829	2.07	0.635	0.409	0.409	1.088	1.314	2.119

Figure 11. Supplemental

Figure 11B . C2C12 myotubes were incubated with 1μM Serdemetan (MDM2 inhibitor) for 24h and 48h . Immunoblot of MDM2

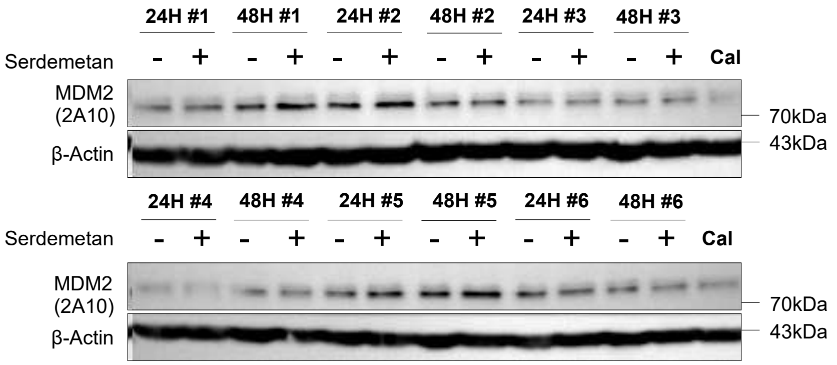

	24H #1		48H #1		24H #2		48H #2		24H #3		48H #3	
Serdemetan (1uM)	-	+	-	+	-	+	-	+	-	+	-	+
MDM2 (2A10)/ β-Actin	0.958	1.169	1.152	1.762	1.221	1.877	1.033	1.174	0.821	0.953	0.749	0.866

	24H #4		48H #4		24H #5		48H #5		24H #6		48H #6	
Serdemetan (1uM)	-	+	-	+	-	+	-	+	-	+	-	+
MDM2 (2A10)/ β-Actin	0.636	0.441	0.84	1.391	1.249	1.825	1.248	1.885	1.115	1.266	0.895	0.88

Figure 11. Supplemental

Figure 11C-11D. C2C12 myotubes were incubated with 1μM Serdemetan (MDM2 inhibitor) for 24h (Fig. 9B) or 48h (Fig. 9C). **C**= Control Sample, **S**=Serdemetan-Treated Sample

Figure 11C. 24H

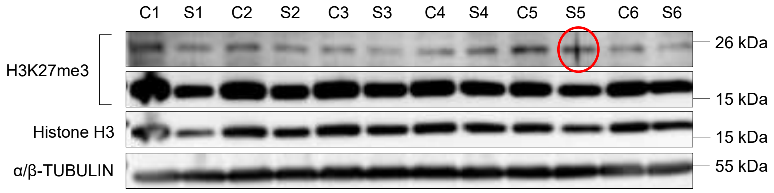

	C1	S1	C2	S2	C3	S3	C4	S4	C5	S5	C6	S6
H3K27me3 (25kDa)/ α/β-TUBULIN	0.083	0.044	0.063	0.044	0.051	0.028	0.054	0.055	0.104	0.072	0.058	0.031

There was a immunoblot issue (vertical line bisecting lane) with the S5 band, therefore, this sample was excluded from data analysis.

Figure 11D. 48H

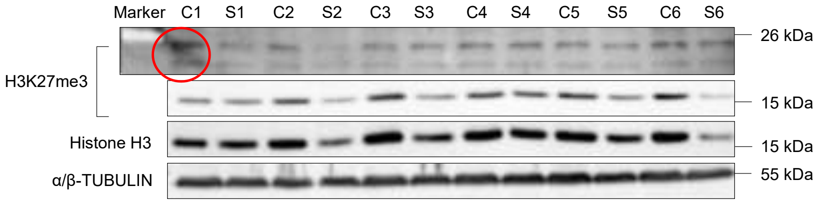

	C1	S1	C2	S2	C3	S3	C4	S4	C5	S5	C6	S6
H3K27me3 (25kDa)/ α/β-TUBULIN	0.147	0.043	0.073	0.023	0.062	0.056	0.076	0.047	0.062	0.051	0.079	0.045

There may have been some spill-over from the molecular weight marker lane into lane C1. This increased the intensity of the detected band. Therefore, this sample was excluded from data analysis.

Figure 11. Supplemental

Figure 11E. *Notch1* mRNA. C2C12 myotubes were incubated with 1μM Serdemetan (MDM2 inhibitor) for 24h and 48h.

Sample ID	*Notch1* Average CT	*Notch1* SD	*Hprt* Average CT	*Hprt* SD	Δ Ct sample	ΔΔ Ct	Fold increase
24H Control #1	27.38	0.23	24.65	0.19	2.72	0.33	1.26
24H Control #2	26.75	0.22	23.89	0.20	2.86	0.19	1.14
24H Control #3	26.91	0.03	24.03	0.06	2.88	0.18	1.13
24H Control #4	27.31	0.08	23.93	0.17	3.38	-0.32	0.80
24H Control #5	27.55	0.05	24.20	0.25	3.35	-0.30	0.81
24H Control #6	27.06	0.10	23.94	0.08	3.13	-0.07	0.95
24H Serdemetan #1	28.26	0.17	25.02	0.01	3.24	-0.18	0.88
24H Serdemetan #2	28.61	0.15	25.02	0.09	3.59	-0.53	0.69
24H Serdemetan #3	27.69	0.06	24.54	0.02	3.15	-0.10	0.93
24H Serdemetan #4	27.48	0.15	24.43	0.20	3.05	0.01	1.01
24H Serdemetan #5	27.59	0.27	24.18	0.23	3.41	-0.36	0.78
24H Serdemetan #6	27.91	0.06	24.47	0.09	3.44	-0.39	0.77
48H Control #1	26.95	0.02	23.92	0.04	3.03	0.12	1.09
48H Control #2	26.86	0.07	23.78	0.10	3.08	0.08	1.05
48H Control #3	26.87	0.04	24.04	0.05	2.83	0.33	1.26
48H Control #4	27.14	0.10	23.84	0.01	3.30	-0.14	0.91
48H Control #5	27.12	0.13	23.79	0.08	3.33	-0.17	0.89
48H Control #6	27.31	0.11	23.93	0.04	3.37	-0.22	0.86
48H Serdemetan #1	27.30	0.00	23.92	0.05	3.38	-0.22	0.86
48H Serdemetan #2	27.44	0.01	24.18	0.03	3.26	-0.10	0.93
48H Serdemetan #3	26.88	0.06	23.86	0.10	3.02	0.14	1.10
48H Serdemetan #4	28.62	0.14	24.64	0.71	3.98	-0.83	0.56
48H Serdemetan #5	27.96	0.15	24.01	0.10	3.95	-0.79	0.58
48H Serdemetan #6	27.89	0.01	23.72	0.10	4.17	-1.02	0.49

Figure 11F. *Notch1* mRNA . C2C12 cells were treated with Serdemetan for 48 hours and then with EPS (90 minutes per day for 4 days in the presence of Serdemetan, SD). DMSO was used as a control vehicle.

Sample ID	Average CT Notch1	Notch1 SD	Average CT Hprt	Hprt SD	Δ Ct sample	ΔΔ Ct	Fold increase
DMSO #1	28.01	0.05	24.86	0.07	3.15	0.19	1.14
DMSO #2	27.64	0.16	24.25	0.08	3.39	-0.05	0.97
DMSO #3	28.10	0.09	24.67	0.03	3.42	-0.08	0.94
DMSO #4	28.03	0.07	24.63	0.05	3.40	-0.06	0.96
DMSO + EPS #1	26.59	0.11	23.69	0.03	2.90	0.44	1.36
DMSO + EPS #2	26.43	0.08	24.38	0.12	2.05	1.29	2.45
DMSO + EPS #3	26.56	0.05	23.89	0.24	2.68	0.66	1.58
DMSO + EPS #4	26.39	0.09	24.06	0.07	2.32	1.02	2.03
SDMN #1	28.11	0.05	23.94	0.02	4.17	-0.83	0.56
SDMN #2	28.45	0.07	24.79	0.10	3.66	-0.32	0.80
SDMN #3	28.80	0.07	24.38	0.13	4.41	-1.07	0.48
SDMN #4	28.77	0.02	24.62	0.06	4.15	-0.81	0.57
SDMN + EPS #1	28.43	0.16	24.06	0.05	4.38	-1.04	0.49
SDMN + EPS #2	28.86	0.07	24.25	0.08	4.61	-1.27	0.41
SDMN + EPS #3	28.67	0.05	24.45	0.02	4.21	-0.87	0.55
SDMN + EPS #4	28.63	0.03	24.40	0.15	4.23	-0.89	0.54

Figure 11G. **Western blot analysis of the impact of Serdemetan treatment on H3K27me3 (n=6)**

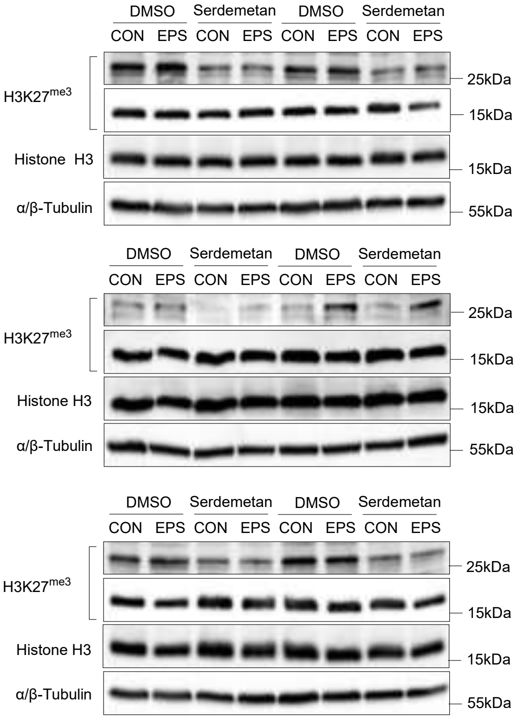

H3K27me3 to Tubulin	25kDa				17kDa			
	CON	EPS	SDMN + CON	SDMN + EPS	CON	EPS	SDMN + CON	SDMN + EPS
1	1.107	1.340	0.933	0.929	0.928	1.096	1.117	1.234
2	0.952	1.105	0.801	0.835	0.950	0.874	1.135	0.667
3	0.959	1.122	0.865	0.906	0.695	0.686	1.148	1.146
4	0.909	1.167	0.890	1.183	1.134	1.040	1.115	1.035
5	1.083	1.517	0.863	0.668	1.081	0.914	1.333	0.920
6	1.038	1.099	0.837	0.894	1.001	0.948	0.952	0.850

H3K27me3 to Histone H3	25kDa				17kDa			
	CON	EPS	SDMN + CON	SDMN + EPS	CON	EPS	SDMN + CON	SDMN + EPS
1	1.231	1.357	0.786	0.777	1.051	1.131	0.960	1.053
2	1.041	1.252	0.745	0.810	1.060	1.009	1.077	0.660
3	1.165	1.496	0.787	0.841	0.876	0.949	1.083	1.104
4	0.813	1.184	0.723	0.991	1.052	1.095	0.941	0.900
5	0.982	1.511	0.680	0.727	0.991	0.920	1.061	1.010
6	1.024	1.196	0.920	0.961	0.997	1.042	1.057	0.923
